# Renalase Protects against Contrast-Induced Nephropathy in Sprague-Dawley Rats

**DOI:** 10.1371/journal.pone.0116583

**Published:** 2015-01-30

**Authors:** Binghui Zhao, Qing Zhao, Junhui Li, Tao Xing, Feng Wang, Niansong Wang

**Affiliations:** 1 Department of Radiology, Tongji University Affiliated Shanghai Tenth People’s Hospital, Shanghai, China; 2 Department of Nephrology and Rheumatology, Shanghai Jiao Tong University Affiliated Sixth People’s Hospital, Shanghai, China; 3 Department of Cardiology, Shanghai Jiao Tong University Affiliated Sixth People’s Hospital, Shanghai, China; 4 St. Vincent’s Hospital, Melbourne, Australia; The University of Manchester, UNITED KINGDOM

## Abstract

**Background:**

Contrast-induced nephropathy (CIN) is the third leading cause of hospital-acquired acute renal failure. Oxidative stress, apoptosis and inflammation play crucial roles in CIN. Renalase is a newly discovered monoamine oxidase from the kidney. We hypothesize that renalase could protect against CIN through anti-oxidation, anti-inflammation and anti-apoptosis pathways.

**Methods:**

We tested our hypothesis *in vivo* with a rat model of Ioversol-induced CIN and *in vitro*. Sprague-Dawley rats were divided into 4 groups (n = 6 per group): control group, Ioversol group (rats subjected to Ioversol-induced CIN), Ioversol plus vehicle group (CIN rats pretreated with vehicle) and Ioversol plus renalase group (CIN rats pretreated with 2 mg/kg recombinant renalase). HK2 cells were treated with Ioversol or H_2_O_2_.

**Results:**

The results showed that pretreatment with renalase attenuated the deterioration of renal function, tubular necrosis, oxidative stress, apoptosis and inflammation (*P<0.05*). Furthermore, renalase protected HK2 cells against the cytotoxicity of Ioversol and suppressed Caspase-3 activity, oxidative stress and apoptosis induced by H_2_O_2_.

**Conclusion:**

Recombinant renalase protected CIN in rats through anti-oxidation, anti-apoptosis and anti-inflammation mechanisms.

## Introduction

With the wide use of iodinated contrast media in diagnostic and interventional procedures, contrast-induced nephropathy (CIN) is becoming a serious problem in clinical practice [1, 2]. The incidence of CIN is not very high in the general population, ranging from 3%–20% [3, 4]. However, the CIN incidence in high-risk populations such as patients with diabetes or chronic kidney disease may increase to 50% [5]. As a result, CIN is the third leading cause of hospital-acquired acute renal failure. CIN results in not only increased medical costs but also worsens patient prognosis and increases the risk of death [6]. There are many risk factors affecting the occurrence of CIN, including age, contrast volume, diabetes, hypertension, and chronic kidney disease [6, 7]. The mechanisms of CIN appear to involve a series of pathological processes such as direct cytotoxicity of contrast, decreased blood flow, renal oxidative stress, apoptosis, and inflammation [8]. Understanding these mechanisms and developing therapeutic interventions for CIN has great clinical value [9].

Renalase, a newly discovered monoamine oxidase enzyme in the kidney, degrades circulating catecholamines and regulates blood pressure and cardiac function [10, 11]. Renalase, a protein made of 342 amino acids with a molecular weight of 37.8 KDa, is closely associated with cardiovascular diseases, diabetes, stroke and chronic kidney disease [12, 13]. Recent advances indicated that renalase exhibited renal protection in a mouse model of ischemia reperfusion injury. Recombinant renalase protein showed anti-oxidant and anti-apoptosis effects *in vivo* and *in vitro* [14, 15]. It is hypothesized that renalase has cytokine-like characteristics [16]. Whether renalase has protective effects against CIN is not known.

We hypothesize that renalase protects against acute kidney injury caused by contrast media. In the present study a rat model of CIN and renal-derived cell lines were employed to test our hypothesis and investigate the involved mechanisms [17, 18].

## Materials and Methods

### Ethical statement

This study was carried out in strict accordance with the recommendations in the Guide for the Care and Use of Laboratory Animals of Tongji University Affiliated Shanghai Tenth People’s Hospital. All animal protocols were approved by the Institutional Animal Care and Use Committee of Tongji University Affiliated Shanghai Tenth People’s Hospital. All surgery was performed under sodium pentobarbital anesthesia, and all efforts were made to minimize suffering.

### Renalase recombinant protein and animals

The recombinant renalase protein was prepared as described previously [19]. Before the experiment, renalase was solubilized in 0.9% saline for administration. Male Sprague-Dawley rats from Shanghai Science Academy animal center weighing 200±20g were housed in individual cages under controlled light (12h dark/12h light cycle) and temperature (20–23°C) conditions. All the rats were allowed to consume standard diet and tap water.

### Rat CIN model and experimental design

This animal study was designed as follows: normal control group (CTL) (n = 6), Ioversol group (Iov) (n = 6), loversol with vehicle group (Iov+Veh) (n = 6) and loversol with recombinant renalase treatment group (Iov+Renalase) (n = 6). The animals were divided into each group by random number table.

In the morning rats in the Iov, Iov+Veh, and Iov+Renalase groups were anesthetized (50 mg/kg pentobarbital, ip), and given a tail vein injection of indomethacin (Sigma, USA) (10mg/kg), followed by Ioversol (Hengrui Corp., China) (3g/kg organically bound iodine) and *N*-nitro-*L*-arginine methyl ester (*L*-NAME) (Sigma, USA). Rats in the Iov+Renalase group also received an intraperitoneal injection of recombinant renalase (2 mg/kg) 30 min prior to inducing CIN. Rats in the Iov+Veh group received the same volume of vehicle at each time point. Animals were kept on warm platforms until wake-up completely. Twenty-four hours later, tissue collection was carried out after anesthetizing (50 mg/kg pentobarbital, ip). The left kidney was harvested for associated analysis. The right kidney was fixed in 10% formalin for histological assessments. Blood samples were collected from abdominal aorta to isolate serum and then stored at -80°C freezer until analyzed. The rats were euthanized by creating pneumothorax at the end of the tissue collection.

### Biochemical markers of renal function

An automatic biochemical analyzer (Hitachi7600, Japan) was employed to measure serum creatinine (SCr) and blood urea nitrogen (BUN) to evaluate the changes of renal function.

### Morphological assessments

The fixed right kidney was embedded in paraffin and then was cut into 3-μm sections. After Hematoxylin-eosin (H.E.) and Periodic acid–Schiff (PAS) staining, the slides were viewed by light microscopy. The renal injury was scored using grading tubular necrosis, loss of brush border, cast formation, and tubular dilatation in 10 randomly chosen, non-overlapping fields. The renal degree of injury was estimated by the following criteria: 0, none; 1, 0–10%; 2, 11–25%; 3, 26–45%; 4, 46–75%; and 5, 76–100%, as described previously [20].

### Renal apoptosis and Caspase-3 activity

A TUNEL staining was employed to compare the renal apoptosis extent in different groups with a commercial kit (#11684817910, Roche, Germany). The caspase-3 activity in renal tissues was measured using a commercial kit (Beyotime, China).

### Oxidative stress in renal tissues

Malondialdehyde (MDA) and superoxide dismutase (SOD) levels in renal tissues were determined using commercial kits to evaluate the balance of oxidative stress and anti-oxidation following the manufacturer’s protocol (Nanjing Jiancheng, China).

### Renal cytokines expression and macrophage infiltration

Quantitative PCR was employed to measure the renal mRNA levels of TNF-α and MCP-1. The primers were as follows: foward: 5’AGGCGCTCCCCAAAAAGA3’, reverse: 5’CCACGAGCGGGAATGAGA3’ (TNF-α); foward 5’CCCCACTCACCTGCTGCTAC3’, reverse: 5’CCTGCTGCTGGTGATTCTCTT5’ (MCP-1). Renal infiltrated macrophages were detected using immunohistochemistry with an anti-F4/80 antibody (#ab74383, Abcam, USA).

### Cytotoxicity of Ioversol to HK2 cells

HK2 cells (ATCC, USA) were cultured in K-SFM at 37°C 5% CO_2_, supplemented with 5 ng/ml human recombinant EGF and 0.05 μg/ml bovine pituitary extract. To investigate the Ioversol’s cytotoxicity, HK2 cells were exposed to 50 mg/mL Ioversol for 24 h. To determine whether recombinant renalase can protect against cell injury, 30 min before the treatment of Ioversol, HK2 cells were preincubated with recombinant renalase at 60µg/ml. The released cytoplasmic enzyme lactate dehydrogenase (LDH) levels in the supernatant were determined using an LDH assay kit (Sigma, St.Louis, USA). Cell apoptosis was measured with a Cell Death Detection kit (#11544675001, Roche, Germany).

### An oxidative stress model of HK2 cells in vitro

HK2 cells were treated with H_2_O_2_ (500 μmol/L) to induce oxidative injuries. To determine whether recombinant renalase can protect against cellular injury, 30 min before the treatment of H_2_O_2_, HK2 cells were pre-incubated with recombinant renalase at 60 µg/ml. Caspase-3 activity (Beyotime, Nantong, China), intracellular reactive oxygen species (ROS) (Cell Biolabs, USA), and cell apoptosis were measured with commercial kit (#11544675001, Roche, Germany).

### Statistical analysis

The software of GraphPad Prism was employed to process the data. All the data were expressed as mean ± standard error. One-way ANOVA with Sidak compensation was used for parametric data and Kruskal-Wallis with Dunn’ compensation for non-parametric data to determine the differences in groups. A value of *P* <0.05 was considered significant.

## Results

### Renalase decreased serum levels of SCr and BUN in CIN rats

Rats subjected to CIN in Iov and Iov+Veh groups presented significant increases of SCr and BUN. However, the increases of SCr and BUN were inhibited in rats treated with recombinant renalase, as shown in Table 1. Furthermore, renalase restored SCr and BUN in part concerned to the control levels. The results indicated that renalase protected against decreased renal function caused by the contrast media Ioversol.

**Table 1:** Renalase decreased levels of serum creatinine, blood urea nitrogen and histological injuries in CIN rats.

	**CTL (n = 6)**	**Iov (n = 6)**	**Iov+Veh (n = 6)**	**Iov+Renalase (n = 6)**
Scr, μmol/L	34.4±1.4	87.1±3.5**	90.8±4.7**	73.6±2.5^##,▲▲^
BUN, mmol/L	6.7±0.5	19.4±1.1**	19.5±1.2**	12.1±1.3^##,▲▲^
Tubular scores	0.8±0.3	3.5±0.2**	3.7±0.4**	2.2±0.3^#,▲^

**P<0.01, vs CTL;

^#^P<0.05, vs Iov+Veh;

^##^P<0.01, vs Iov+Veh;

^▲^P<0.05, vs CTL;

^▲▲^P<0.01, vs CTL (One-way ANOVA with Sidak compensation)

### Renalase ameliorated renal histological damage

**Figure 1:**
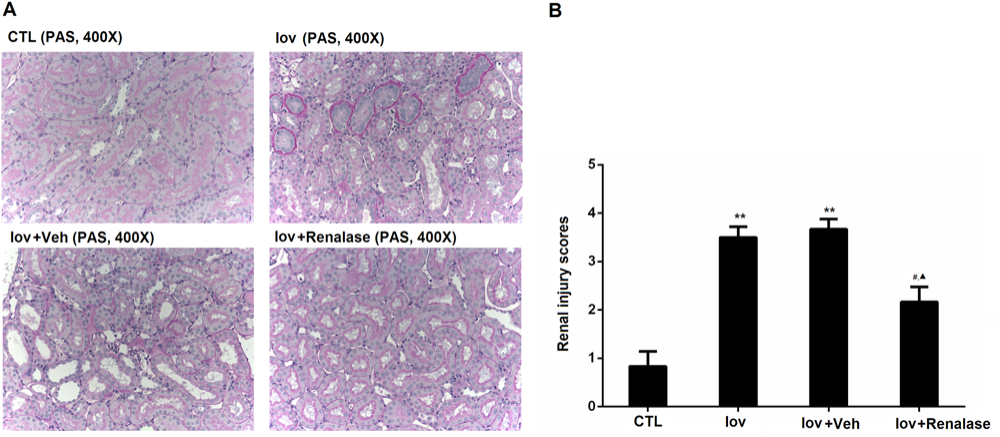
Renalase ameliorated renal histological damage. A, representative renal sections from normal control, Ioversol, Ioversol+Vehicle, and Ioversol+renalase groups (PAS staining, 400x). B, tubular injury scoring and quantitative analysis. Animal number in each group is 6. **P<0.01, vs CTL group; #P<0.05, vs Veh group; ▲P<0.05, vs CTL (Kruskal-Wallis with Dunn’ compensation).

The pathological findings in the four groups were summarized in Fig. 1. In the kidney sections of Iov and Iov+Veh groups, the tubular detachment, foamy degeneration, and necrosis can be observed. The morphological alterations in the renalase treatment group were less severe than that in the Iov and Iov+Veh groups. The renal injury scores in the renalase treatment group were reduced compared to the Iov and Iov+Veh groups (Fig. 1 and Table 1), which was consistent with the changes of renal function. However, renalase pretreatment restored the histological damage partialy.

### Renalase inhibited renal apoptosis induced by Contrast

**Figure 2:**
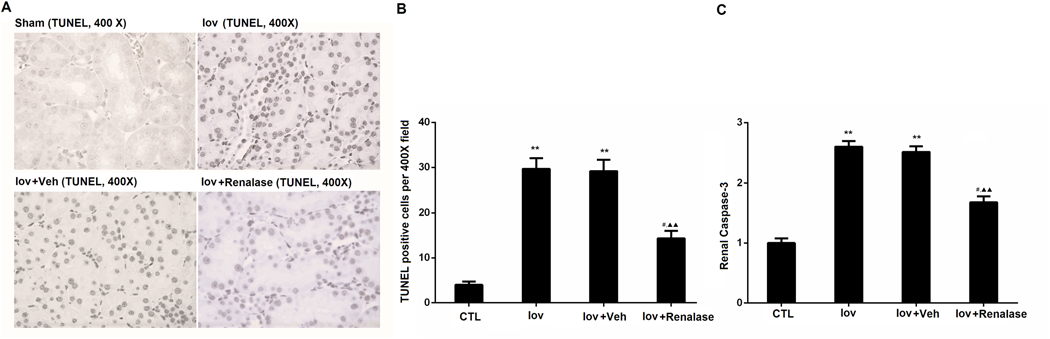
Renalase inhibited renal apoptosis induced by Contrast. A, representative renal sections from normal control, Ioversol, Ioversol+vehicle, and Ioversol+renalase groups (TUNEL staining, 400x). B, quantitative analysis of renal apoptosis. C, renal Capase-3 activity. Animal number in each group is 6. **P<0.01, vs CTL group; #P<0.05, vs Veh group; ▲▲P<0.01, vs CTL (One-way ANOVA with Sidak compensation for the analysis of apoptosis, Kruskal-Wallis with Dunn’ compensation for Caspase-3).

Quantitative analysis revealed that the TUNEL-positive cells (per 400X fields) were significantly less abundant in the Iov+renalase group (16.7±3.0) than that in the Iov+Veh (29.1±2.5) groups (P<0.05), as shown in Fig. 2. In comparison, renalase preconditioning reduced the tubular apoptosis in rats with CIN (Fig. 2A and 2B). Compared with the CTL group, Capse-3 activity increased significantly in Iov (2.57±0.10 fold) and Ivo+Veh (2.51±0.09 fold) groups (*P*<0.01, respectively). Moreover, renalase pretreatment in CIN+Renalase group exhibited decreased Caspase-3 activity (1.91±0.15 fold) compared with Iov+Veh group (P<0.05) (Fig. 2C). In addition, renalase pretreatment inhibited renal apoptosis and Caspase-3 activity partialy as shown in Fig. 2.

### Renalase reduced oxidative stress and renal inflammmation

Renal levels of MDA were elevated in the Iov and Iov+Veh groups compared with the normal control group (*P*<0.05) as shown in Table 2. Moreover, lower renal levels of SOD were observed in the Iov and Iov+Veh groups. Pretreatment with recombinant renalase decreased renal MDA levels and increased renal SOD levels dramatically, which demonstrated that renalase could reduce oxidative stress from both directions in rats with CIN. In addition, renalase decreased renal TNF-α and MCP-1 levels as well as infiltrated macrophages (Table 2). Furthermore, the variable levels in Iov+Renalase were not restored to the control levels. This indicates renalase can reduce renal oxidative stress and inflammation in CIN rats partialy.

**Table 2:** Renalase reduced oxidative stress and renal inflammation in CIN rats.

	**CTL (n = 6)**	**Iov (n = 6)**	**Iov+Veh (n = 6)**	**Iov+Renalase (n = 6)**
Renal MDA, nmol/mg	6.7±0.5	14.7±0.7**	15.5±0.6**	9.5±0.5^##,▲▲^
Renal SOD, U/mg	56.2±4.1	24.2±2.6**	21.8±1.5**	35.3±4.7^#,▲▲^
TNF-α mRNA, fold	1.00±0.05	2.43±0.10*	2.50±0.17*	1.32±0.03^#,▲▲^
MCP-1 mRNA, fold	1.00±0.03	1.92±0.12*	1.87±0.11*	1.41±0.07^#,▲▲^
Infiltrated macrophage, per 400X field	6.1±0.3	16.2±0.9**	16.5±1.0**	12.4±1.1^#,▲▲^

*P<0.05, vs CTL;

**P<0.01, vs CTL;

^#^P<0.05, vs Iov+Veh;

^##^P<0.01, vs Iov+Veh;

^▲▲^P<0.01, vs CTL (One-way ANOVA with Sidak compensation for the analysis of MDA, SOD and macrophage, Kruskal-Wallis with Dunn’ compensation for TNF-α and MCP-1)

### Renalase protected against Ioversol’s cytotoxicity, oxidative stress and apoptosis in vitro

**Figure 3:**
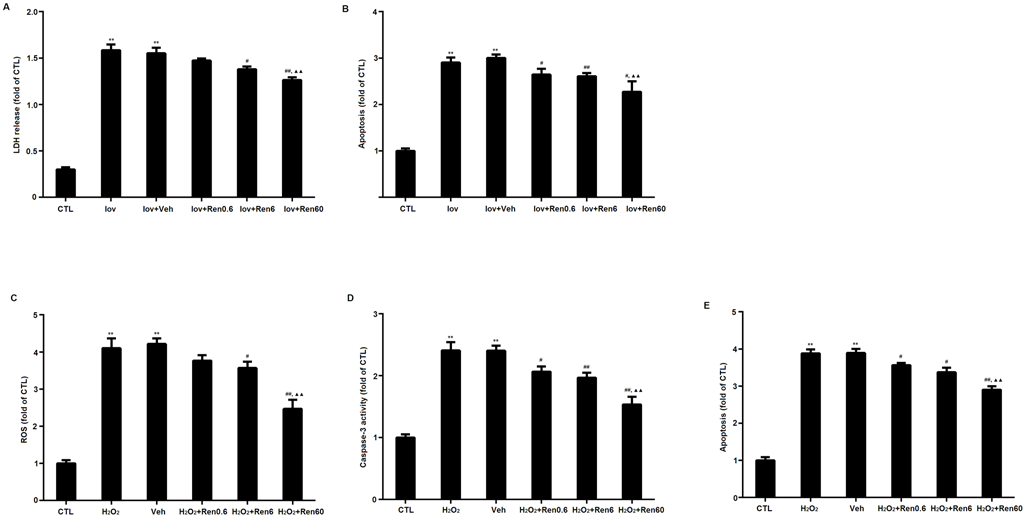
Renalase protected against oxidative stress and apoptosis in vivo. A, LDH release of HK2 cells treated with Ioversol. B, apoptosis of HK2 cells treated with Ioversol. C, ROS levels of HK2 cells treated with H_2_O_2_. D, Caspase-3 activities of HK2 cells treated with H_2_O_2_. E, apoptosis of HK2 cells treated with H_2_O_2_. Iov, ioversol; Veh, Vehicle; Ren0.6, renalase 0.6 µg/ml; Ren6, renalase 6 µg/ml; Ren60, renalase 60µg/ml. The number of repeated wells is 6 in each group. **P<0.01, vs CTL group; #P<0.05, ##P<0.01 vs Veh group; ^▲▲^P<0.01, vs CTL (Kruskal-Wallis with Dunn’ compensation).

*In vitro*, Ioversol caused a pronounced increase in LDH release and apoptosis in HK2 cells, which indicated that Ioversol could induce cytotoxicity to tubular cells directly. Renalase pretreatment provided protective effects against Ioversol’s cytotoxicity (Fig. 3A and 3B). ROS and Caspase-3 levels elevated in HK2 cells treated with H_2_O_2_ (500 μmol/L), and apoptosis levels also increased. Moreover, the protective effects of renalase were dose-related as shown in Fig. 3. However, the renalase pretreatment restored the variable in part concerned to control levels. The results suggested that renalase pretreatment decreased the levels of ROS, Caspase-3 and apoptosis (Fig. 3C, D and E).

## Discussion

Contrast media is used widely in many diagnostic and interventional procedures, and is necessary component in modern medical technology. Unfortunately this usage is linked to an increase in hospital-acquired acute renal failure as CIN becomes more and more common. Although many studies focus on the prevention of CIN, there is not a particularly promising therapy yet [1]. Finding a preventative tool against CIN is urgent and important clinical goal, especially considering the rate of growth of the at-risk patient population.

This study presented for the first time that recombinant renalase pretreatment before the administration of contrast media could prevent the CIN in rats. Recent advances showed that the mechanisms of CIN involve not only the toxicity of contrast media, but also renal ischemia, oxidative stress, and cell apoptosis [21]. Our results showed that recombinant renalase ameliorated the renal injury, tubular necrosis, renal inflammation, and the deterioration of renal function in a rat CIN model. Furthermore, renalase exhibited a direct protection against contrast’s cytotoxicity *in vitro*. Renalase reduced oxidative stress and suppressed Caspase-3 activity *in vivo* and in the H_2_O_2_-induced oxidative cell model.

Oxidative stress plays a crucial role in the mechanisms of CIN. After the administration of contrast media, ROS enhance, leading to lipid peroxidation and cytotoxic damage. Free radicals react with nitric oxide to produce peroxynitrite, reducing the bioavailability of nitric oxide, thereby increasing tissue damage. Reactive species exerts its oxidative effects on the sulphydrylic groups and aromatic rings of proteins, cellular membrane lipids and nucleic acids [22]. MDA is one end product of lipid peroxidation of membrane polyunsaturated fatty acids and one indicator of oxidative damage [22]. The identification of drugs that can scavenge ROS has been a major focus in the CIN prevention research [18, 22]. In this study we observed pronounced increases in renal MDA and decreases in renal SOD in rats with CIN. However, recombinant renalase preconditioned rats expressed lower renal MDA and higher renal SOD, as well as having reduced kidney injury. In addition, renalase decreased ROS generation in an oxidative stress model *in vitro*. We speculate that renalase decreases contrast-induced explosion of MDA, therefore ameliorating the renal injury. Thus, renalase exhibited anti-oxidative effects in this study, which was consistent with a previous report [15].

Apoptosis is involved in the mechanisms of acute kidney injury including CIN [23, 24]. It has been reported that suppression of the apoptosis pathways may attenuate pathological alterations in CIN. Our data showed severe histological injury in the CIN kidney. Moreover, increased levels of Caspase-3 activity and apoptosis were observed in CIN rats or HK2 cells, which were partly reversed by renalase *in vivo* and *in vitro*. This suggests that the renoprotection of renalase against CIN could be associated with its anti-apoptotic effects.

In addition, inflammation is another pivotal mechanism of CIN [25, 26]. This study showed the pronounced contrast media-induced increase of renal TNF-α and MCP-1 was reduced by renalase. It is known that MCP-1 contributes to the tissue infiltration of macrophages [27]. In this study the renal infiltrated macrophages were suppressed in CIN rats treated with renalase. Thus, renalase exhibited anti-inflammatory effects in the CIN model. The limitation of this study is that the detailed pathways about renalase’s renoprotection were not investigated. Thus, the detailed molecular mechanisms and pathways involved in the renoprotective effects of renalase should be further investigated.

In conclusion, this study has showed that pretreatment with renalase exhibited protection against CIN via anti-oxidation, anti-apoptosis and anti-inflammation mechanisms. These findings indicated that renalase could be a potential therapeutic intervention to prevent CIN in the future.
